# Abnormal ore pressure mechanism of working face under the influence of overlying concentrated coal pillar

**DOI:** 10.1038/s41598-024-51148-x

**Published:** 2024-01-05

**Authors:** Cao Zhengzheng, Sun Qiang, Li Zhenhua, Du Feng

**Affiliations:** 1 International Joint Research Laboratory of Henan Province for Underground Space Development and Disaster Prevention, School of Civil Engineering, Henan Polytechnic University, Jiaozuo, 454000 Henan China; 2 Collaborative Innovation Center of Coal Work Safety and Clean High Efficiency Utilization, Jiaozuo, 454000 Henan China; 3 Henan Mine Water Disaster Prevention and Control and Water Resources Utilization Engineering Technology Research Center, Henan Polytechnic University, Jiaozuo, 454000 Henan China

**Keywords:** Coal, Civil engineering

## Abstract

Shenfu Dongsheng coal field is a cross-century energy base which is developed and constructed in China. In recent years, some mines have successively entered to the coal seam of the second layer. Due to the reasons of early mining, many coal pillars are left in the coal seam of the first layer, resulting in the phenomenon of strong ore pressure in the mining range before and after the coal pillar in the lower coal seam and even causing the buckling accident. In order to solve such safety problems, this paper takes the 22,307 working face in Bulianta coal mine as the research object, adopts physical similarity simulation experiment and theoretical analysis to systematically study the overlying rock characteristics and abnormal ore pressure manifestation mechanism of shallow and close coal seam in different working stages. The results show that the roof overburden of the key layer in the lower group bends and sinks when the coal pillar is mined, resulting in the activation and instability of the “masonry beam” structure formed by the roof of the upper coal seam. When the coal pillar is discharged, the residual concentrated coal pillar and the room type coal pillar are unstable under the action of high supporting stress, resulting in shear failure of the inter-layer rock in the upper part of 22,307 working face, causing the strong dynamic pressure of the working face to appear and then leading to the buckling accident. The working resistance of the support in each stage is obtained by establishing the structure diagram of the overlying rock under each stage and the corresponding mechanical structure model. Finally, the working resistance required by the support in the mining stage under the goaf is 16,692.6 kN, the working resistance required by the support in the coal pillar stage is 19,692.6 kN, the working resistance required by the support in the mining stage under the concentrated coal pillar is 13,150.6 kN, and the working resistance required by the support in the coal pillar stage is 19,215.6 kN.

## Introduction

China is a large resource country, with a fundamental energy composition that is rich in coal, poor in oil, and low in gas, implying that coal resource plays a vital role in Chinese energy consumption^1–3^. The Shenfu Dongsheng coal field is located at the confluence of the provinces of Inner Mongolia, Shaanxi, and Shanxi in China, is a cross-century energy base, with confirmed coal resource reserves in the hundreds of billions of tons. In recent years, with the increasing intensity and depth of coal mining, the mining conditions have changed from simply to complexity, and the intense mine pressure in the working face beneath the residual coal pillar has produced an increasing number of safety issues^4–6^. When mining in the lower working face, there are severe pressure phenomena such as short-term and significant shrinkage of the support pillars or the support being crushed during the entry and exit of the remaining coal pillars, which seriously threaten the safety and efficient production in coal mine^7–9^. Therefore, it is necessary to study the mechanism of abnormal mineral pressure manifestation when passing over overlying concentrated coal pillars under shallow and close mining conditions.

At present, many scholars have studied the problem of mine pressure manifestation in the mining face of shallow buried coal seams^10–14^. In the 1980s, Qian Minggao^15^ put forward the theory of “masonry beam”, which realizes the leap from qualitative to quantitative development in the study of mine pressure. The broken rock blocks squeeze each other to form a horizontal force, and the friction between the rock blocks generates, forming a fractured body beam structure that looks like a beam and is essentially an arch, which is called "masonry beam". Generally speaking, the key layer is the main bearing layer, which can bear part of the weight of the upper rock layer in the form of a “plate” or “beam” structure before breaking, and form a masonry beam structure after breaking. The theory of “masonry beam” points out that in the overlying rock layer of the working surface, there are some rock layers of relatively large thickness and strength, which are called the key layers bearing the load of the overlying soft rock and controlling the movement of the rock layers. Based on the mechanical analysis of the “masonry beam” structure, Miao Xiexing, He Fulian and others scholars^16–18^ further simplified the key blocks of the “masonry beam” as a “three-hinged arch” structure, established the “S–R” theory (sliding instability and rotation instability), analyzed the stability of the three-hinged arch, and proposed the critical conditions of sliding instability and rotating deformation instability. Song Zhenqi^19–21^ put forward the “transfer rock beam hypothesis”, which holds that the interlocking between fractured rock blocks can transfer the force to the front of the coal wall and the gangue in the goaf, so the force during the movement of the rock beam does not need to be borne by the bracket in its entirety; the bracket assumption of the size of the force of the rock beam is determined by the requirements of the control of its movement. Xu Jialin, Zhu Weibing and other scholars^22^ proposed the discrimination method of overburden structure for the conditions of shallow buried coal seam in Shendong mine area, and divided the type of key layer structure into single key layer and multi-layer key layer structure. Due to the excessive thickness of roof loading layer in Shendong mine area, the key block of masonry beam structure is prone to slip and instability, which is the fundamental reason why the working face of single key layer structure in shallow buried coal seam is prone to trigger mining disasters such as step sinking and strong dynamic pressure.

Domestic and foreign scholars have conducted a series of researches on the phenomena of step-down and strong dynamic pressure when the shallow buried large-height working face passes over the overlying concentrated coal pillars, from the law of overlying rock transportation and the structure of overlying rock in the quarry space. It is obvious that the research achievements of the phenomena of step-down and strong dynamic pressure have been obtained by laboratory experiment, numerical simulation, and theoretical analysis. However, the abnormal ore pressure mechanism of working face under the influence of overlying concentrated coal pillar in the Shendong mining area has not been carried out at present. Therefore, in order to better understand the ground pressure of the working face passing the coal pillar in Shendong mining area, this paper studies the ground pressure behavior law in Bulianta 22,307 working face when it has mined the concentrated coal pillar, and further explains the reasons for the phenomenon that the support pillar shrinks greatly and the strong ground pressure when it enters and exits the overlying concentrated coal pillar.

## A similarity simulation experiment of overburden movement

### Physical similarity simulation model design

The experiment is designed based on the geological conditions under repetitive mining conditions at the 22,307 working face in the Bulianta coal mine. Bulianta coal mine, developed and constructed by Shendong Coal Group, is located in Wulan Mulun Town, Yijin Horo Banner, Ordos City, Inner Mongolia. It is currently the world's largest single mine with an approved production capacity of 28 million tons/year. The dip angle of 22,307 working face is 1 ~ 3°, the average dip angle is 2°, the average thickness of 2–2 coal seam is 7.25 m, the length of working face is 301 m, the length of inclined advance is 4954 m, the design mining height is 6.8 m, and the goaf is treated by the collapsed method.

The physical similarity simulation of mine pressure satisfies the similarity conditions, containing geometric similarity, kinematic similarity, dynamic similarity, stress similarity, external force similarity, stress–strain relationship similarity, strength curve similarity, and time characteristic similarity. According to the laboratory conditions and other aspects of the comprehensive consideration to determine the geometric similarity ratio of the model, that is, the model and the actual size ratio C_l_ = 1:100; the similarity ratio of the capacity C_γ_ = 1:1.5; model time ratio is 1:10.

According to the exploration and research of physical similarity simulation experiments at home and abroad, the selection of similarity materials meet the following requirements.

1. The mechanical index is stable and does not change the mechanical properties due to changes in atmospheric temperature and humidity.
2. After changing the ratio, the mechanical index of the material can be greatly changed, so as to facilitate the choice of use.
3. Easy to make, short solidification time, low cost, rich sources, it is best to reuse.
4. It is easy to set the measuring sensor, and there is no dust and poison that damage the health of workers during the production process.

According to the specific conditions of the laboratory, similarity materials are selected as follows.

1. *Aggregate* river sand, particle size less than 2 mm.
2. *Cementing material* calcium carbonate, gypsum retarder, borax.
3. *Layered material* mica sheet.

**Figure 1:**
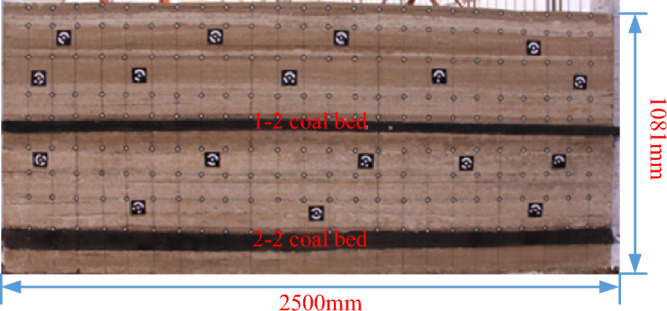
Physical similarity simulation model.

According to the mining and geological conditions in 22,307 working face, the conditions of physical similarity simulation test, and the lithology of the roof and floor of the coal seam, and the inter-layer spacing of No.1–2 and No.2–2 coal seams are set. This physical similarity simulation experiment uses a plane stress model test bench with a length, width, and height of 2500 mm, 200 mm, and 1300 mm, respectively. The total thickness of the experimental simulation is 108.1 m, that is, a total of 25 layers from the 2–2 coal seam floor to the surface, of which the cumulative thickness of the floor is 8.8 m, the thickness of the 2–2 coal is 7.5 m, and the cumulative thickness of the roof strata is 91.8 m. For the layer with relatively large thickness of rock layer, the layer is subdivided according to the thickness of 2 cm, and the model is laid layer by layer. The mica sheet is laid between each layer to simulate the inter-layer joint structure. Since this experiment can be directly simulated to the surface according to similarity conditions, no external load is applied during the experiment. The laid model is shown in Fig. 1.

### Layout of measurement points and excavation plan

When the experimental model reaches the excavation humidity, the observation line is arranged on the model surface, the observation line adopts the cross-crossing design, the interval between the longitudinal observation line is 0.1 m, the transverse observation line is arranged in the main rock layer, and the intersection of the transverse and longitudinal observation lines is arranged with reflective measurement points.

**Figure 2:**
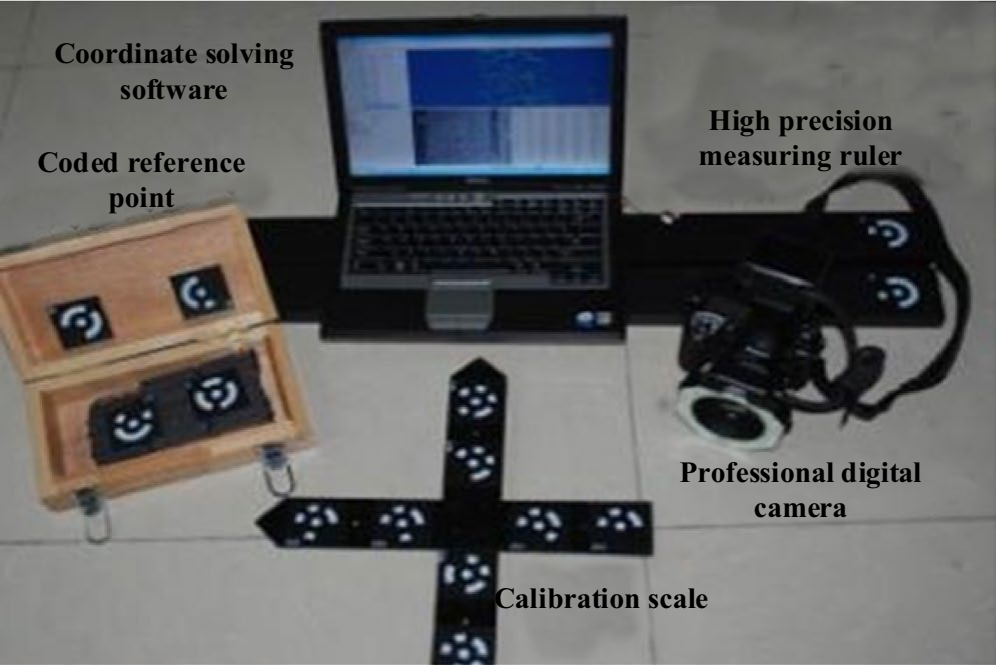
3D optical photogrammetry system.

The experimental displacement observation uses a three-dimensional optical photogrammetry system, shown in Fig. 2. The system can measure the three-dimensional coordinates of the object surface more accurately by data processing through the splicing technology of object point cloud. Photographs are taken before each excavation, and the system can calculate the displacement change of each measurement point relative to the initial state of the model during each excavation.

**Figure 3:**
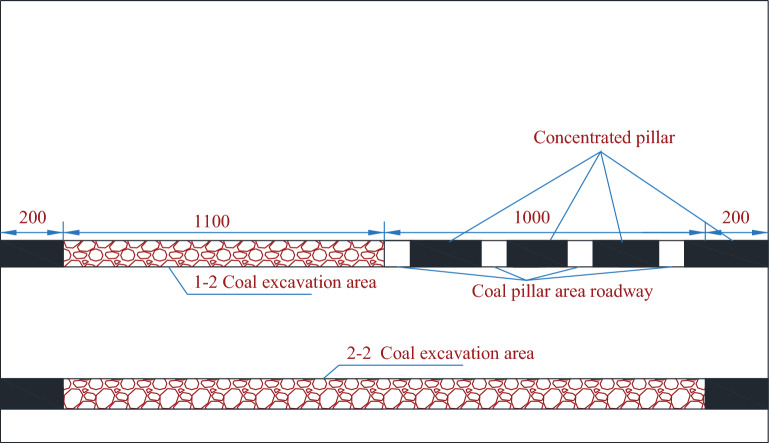
Schematic diagram of excavation area.

This physical similarity simulation experiment simulates the mining process of the 2–2 coal seam working face from the 1–2 coal mining area to the 1–2 coal pillar, so the excavation is roughly divided into three stages, and the schematic diagram of the excavation area is shown in Fig. 3.

1. Excavate the roadway in the 1–2 coal pillar area. After the excavation is completed, the main withdrawal channel is filled with plastic foam material to ensure the integrity of the roadway section during the excavation of 1–2 coal.
2. Excavate the 1–2 coal seams, and the excavation area is shown in the upper shaded part in Fig. 3. Starting from the left side of the model, excavate 8 cm each time with an interval of 30 min. and the observation record is made during the excavation.
3. After the excavation of 1–2 coal seam is completed, the model is left for 1 day, waiting for the 1–2 coal roof to stabilize. After that, the excavation of 2–2 coal seam is carried out, the thickness of the coal seam simulated by the model is 7.5 m, and the thickness of the excavation is taken as the actual mining height of 6.8 m. The excavation starts from the left side of the model, and the excavation is carried out with 8 cm each time, and the interval of each excavation is 30 min. During the excavation, the process of the overburden rock movement and the development of the cracks, as well as the spanning fall and the separation of the layers are recorded, and the photographs are taken before the excavation.

### Result analysis of physical similarity simulation experiment

#### Initial mining of overburden structures

**Figure 4:**
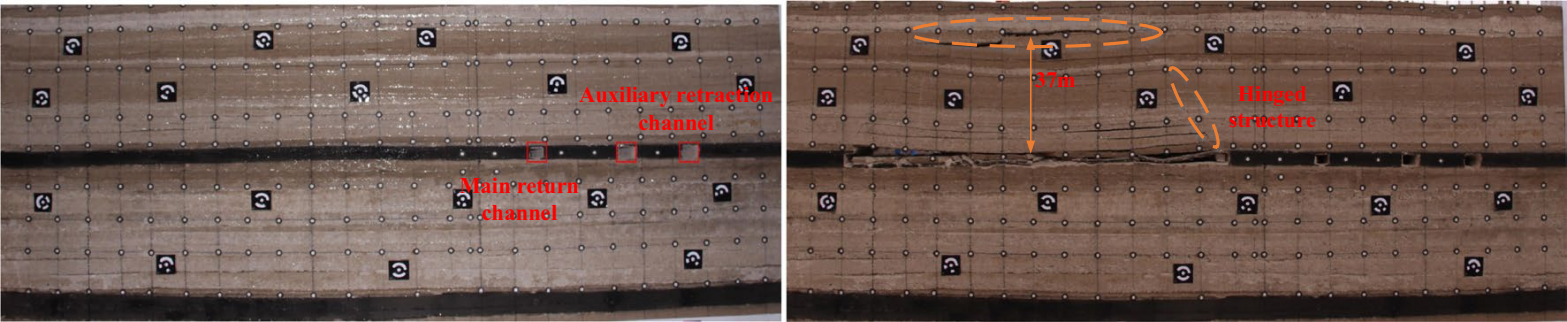
Tunnel excavation of 1–2 coal seam.

As shown in Fig. 4, three roadways are excavated in the 1–2 coal seam in order at 120 m from the left side of the model, the width of the roadway is 5.5 m, and the height is the thickness of the coal seam. After the roadway excavation is completed to form the 1–2 coal pillar, the width of the coal pillar from left to right in order of 25 m, 23 m, 14 m. The 20 m is reserved on the left side of 1–2 coal seam to protect the coal pillar, and excavating from left to right in sequence, with each excavation of 10 m and a time interval of 1 h. Excavating continuously for 110 m to reach the position of the main withdrawal channel. At this time, the main key layer of the coal seam roof forms a masonry hinge structure, and the hinge point at the boundary of the coal pillar is located above the main withdrawal channel.

#### Repeated mining of overburden structures

After the excavation of 1–2 coal seam, the model is static for 12 h, and then excavate 2–2 coal seam, keep 20 m to protect the coal pillar on the left side of 2–2 coal seam; and excavate from the left side of the model to the right side in turn, each time to excavate 2 m, with a time interval of 15 min.

**Figure 5:**
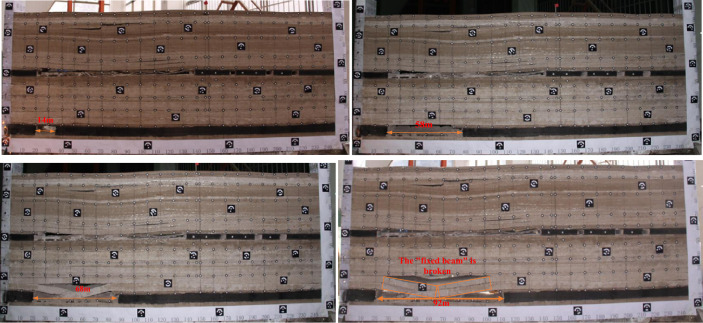
2–2 Coal seam excavation before passing the upper coal pillar.

As shown in Fig. 5, the excavation of 2–2 coal seam is 14 m, at this time the direct top does not collapse. With the advancement of the working face, the gap between the direct top and the basic top is getting bigger and bigger. When the working face advances to 58 m, the direct top collapses, and the direct top collapse distance is 47 m. With the continued excavation of the 2–2 coal seam, the direct top begins to collapse by the action of gravity; when the working face advances to 68 m, the main key layer of the lowest stratum collapses. During the advancement of the working face from 68 to 92 m, the direct roof and the lowest sub-layer of the main key layer collapses with the mining span, and the second sub-layer of the main key layer forms a solidly supported beam structure, and the second sub-layer of the main key layer fractures when it advances to 92 m.

**Figure 6:**
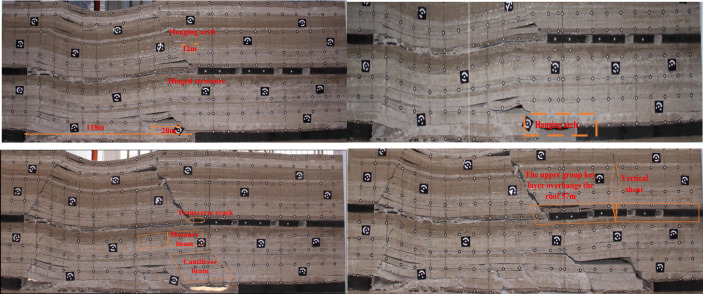
2–2 coal seam excavation passing coal pillar.

As shown in Fig. 6, when the working face advances to 118 m, the working face is located below the main retraction channel of 1–2 coal seam. The lower part of the key layer of the lower group has broken and collapsed, forming a “cantilever beam” structure, and the upper part of the key layer of the lower group has formed an articulated structure with the rock layer that has not collapsed in front of it, and at this time, it can be considered that the upper part of the key layer of the lower group has formed a “masonry beam” structure. The top plate of 1–2 coal seam of the upper group is activated to sink due to the bending and sinking of the lower rock layer, which causes the rotational deformation of the lower rock layer to increase, and at this time, the key layer of the upper group forms the structure of “cantilever beam”, with a cantilever length of 12 m.

As the working face continues to excavate, the transverse fissure between the direct top rock layer of the overlying centralized coal pillar and the key layer of the upper group gradually increases; and when the excavation reaches 180 m, the “cantilever beam” structure of the key layer of the upper group reaches fracture, and at this time, the broken distance of the “cantilever beam” is 57 m. The transmission influence range of the supporting stress exceeds 50 m, which affects the stability of the residual centralized coal pillars and the room columns of the room mining area close to the centralized coal pillars. When the coal pillar is discharged, the residual concentrated coal pillar and the room coal pillar are destabilized together under the action of high supporting stress, and the upper group of key layers fracture in the inner side of the coal pillar of the room pillar, and the long overhanging top and the rock layer controlled by its upper part rotates and sinks synchronously, resulting in the shear damage of the inter-layer rock layer in the upper part of the 2–2 coal seam, and the upper and lower two groups of key layer combinations break and come to be pressurized, causing the working face to be strong and dynamic pressure to be visible and then triggering the pressurized frame accident.

## Structural form and stability analysis of quarry overburden

Based on physical similarity simulation test results, it is known that the thickness of the direct roof of the 22,307 working face is too small, and it can not completely fill the void area after the direct roof collapses, so the lower part of the key layer of the lower group forms a "cantilever beam" structure, and the upper part of the layer forms a "masonry beam" structure. When mining into the overlying centralized coal pillar, the overlying rock of the lower key layer bends and sinks, resulting in the activation and destabilization of the "masonry beam" structure formed in the roof of the 1–2 coal seam. When the coal pillar comes out of the coal pillar, the residual centralized coal pillar is destabilized under the action of the high supporting stress. However, the relationship between the structure formed by the key layer of overburden and the support at each stage of mining back to the face is unclear, so the change rule of overburden in the 2–2 coal seam of the 22,307 face from the mining hollow area to the overburden centralized coal pillar is studied, and the law of the manifestation of the mining pressure by establishing the corresponding mechanical model is analyzed.

### Mechanical and structural analysis of key layers of lower group

#### Thick key layer breaking characteristics

**Figure 7:**
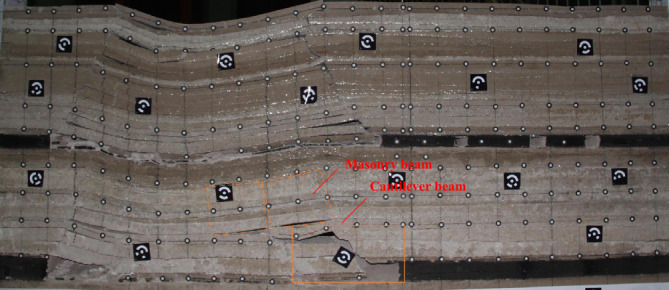
Combined structure of cantilever beam-masonry beam.

According to the results of physical similarity simulation experiment, when the 2–2 coal seam is mined, the thick key layer has the phenomenon of stratified collapse. As the working face advances forward, the lowest stratum of the key layer breaks down and falls into the goaf, unable to transmit the horizontal force forward, forming a “cantilever beam”, while the lower stratum that has already collapsed on top of it forms a “masonry beam” structure at the end, shown in Fig. 7.

#### Thickness determination of the stratified collapse in lower giant critical layer

When the thickness of the intermediate layer between the coal seam and the key layer is small, the basic top of the working face near the coal seam is easy to form stratified breaking characteristics. When the 22,307 working face in Bulianda coal mine is mined, a layer of medium-grained sandstone with a thickness of 29.88 m above the working face is judged as the key layer. When the working face is mined with a large mining height, the basic top rock crosses into the goaf. This part of the rock layer does not function as a basic top^23–25^.

The height of the overlying key layer acting as a fallout zone is

1 $$ h = \frac{M}{{k_{p} - 1}} - h_{z} $$

where *M* is the mining height of the coal seam; *k*_*p*_ is the coefficient of expansion of the rock layer in the collapse zone, generally 1.25 ~ 1.5; *h*_*z*_ is the thickness of the direct top.

**Figure 8:**
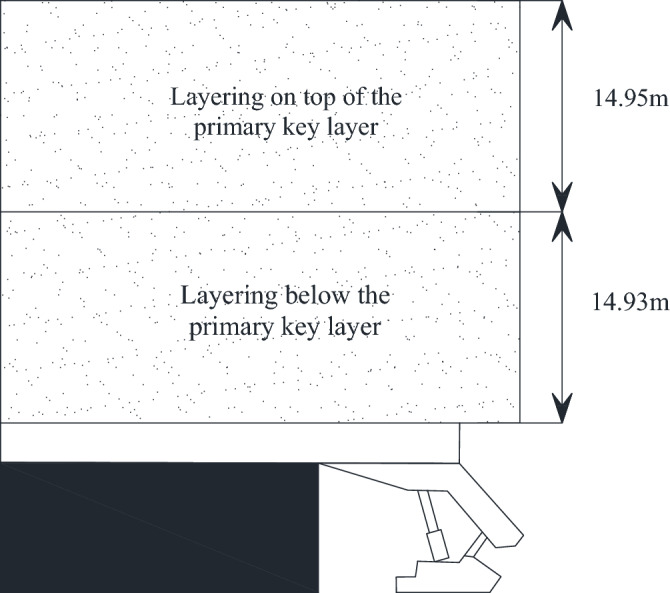
Structural characteristics of key layers in 22,307 working face.

The mining height is 6.8 m, the expansion coefficient is 1.407, the direct top is 1.76 m, and the height of the fallout zone is 16.69 m, so the thickness of basic top is 14.95 m. Then *i* = 14.95/19.3 = 0.77, so the lower split level of 14.93 m forms a cantilever beam structure, and the upper split level forms a masonry beam structure. As illustrated in Fig. 8.

### Structural mechanical model of single key roof stratum

**Figure 9:**
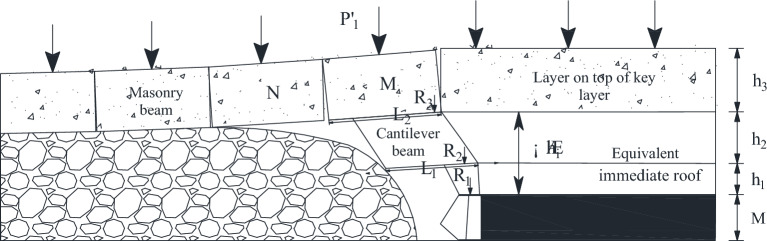
Structural drawing of “masonry beam” on the upper layer of key layer.

When 2–2 coal seam is excavated, the mechanical model of a single key layer can be temporarily established. According to the above analysis, when 2–2 coal seam is excavated, the main key layer collapses in layers, forming a "cantilever beam" structure under the key layer and a “masonry beam” structure above the key layer. The structure diagram of the layered "masonry beam" on the key layer is shown in Fig. 9.

### Masonry beam structural analysis

**Figure 10:**
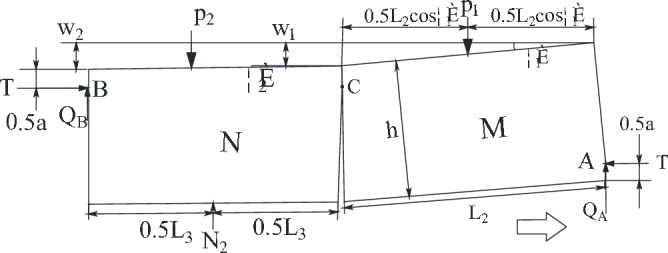
Stress model of key block of "masonry beam".

According to the previous analysis, the "cantilever beam" forms when the lowest stratum of the key layer breaks down in the hollow area and could not transmit the horizontal force forward. Therefore, the structure of "masonry beam" formed in the upper part of the key layer analyzed. The key block force model of "masonry beam" is established in Fig. 10.

In Fig. 10, θ_2_ is small and can be set as 0. The sinking amount of rock mass N, W_1_, can be determined according to the thickness of the direct roof, the mining height, and the coefficient of crushing and expansion, which is determined by the following Eq. (2).

2 $$ W_{1} = M - \left( {k_{p} - 1} \right)\sum h $$

Based on the geometrical relationship of rock block slewing in Fig. 10, the height of the extrusion contact surface of rock blocks M and N can be determined as

3 $$ a = \frac{1}{2}\left( {h - L_{2} \sin \theta_{1} } \right) $$

Since there is a plastic hinge relationship between the block contacts, the point of action of the horizontal force is located at 0.5a.

Due to the basic roof periodic breaking force conditions are basically the same, the periodic weighting step is roughly equal, so in the analysis of short masonry beam key block can be in accordance with the *L*_2_ = *L*_3_ = *L*, take A point bending moment ∑*M*_A_ = 0, B point bending moment ∑*M*_C_ = 0, ∑*F*_*y*_ = 0, and approximate *p*_2_ = *N*_2_, thus

4 $$ \sum {M_{A} } = Q_{B} (L\cos \theta_{1} + h\sin \theta_{1} + L) - P_{1} (0.5L\cos \theta_{1} + h\sin \theta_{1} ) + T(h - a - W_{2} ) = 0 $$

5 $$ \sum {Mc = \frac{1}{2}P_{2} L} + T(W_{2} - W_{1} ) - Q_{B} L - \frac{1}{2}N_{2} L = 0 $$

6 $$ Q_{A} + Q_{B} = P_{1} $$

By geometric relations, *W*_1_ = *L*sinθ_1_,*W*_2_ = *L*(sinθ_1_ + sinθ_2_), Basic roof rock block degree *i*, from formula (4), formula (5), and formula (6):

7 $$ T = \frac{{4i\sin \theta_{1} + 2\cos \theta_{1} }}{{2i + 2\sin \theta_{1} (\cos \theta_{1} - 2)}}P_{1} $$

8 $$ Q_{A} = \frac{{4i - 3\sin \theta_{1} }}{{4i + 2\sin \theta_{1} (\cos \theta_{1} - 2)}}P_{1} $$

According to the literature "S–R" stability analysis, when the thickness of the load layer is less than 180 m, the "short masonry beam" is difficult to rotary instability, so this focus on analyzing the "short masonry beam" slip instability conditions. In order to prevent the "short masonry beam" slip instability, the condition need be met,

9 $$ T\tan \delta + R_{3} \ge Q_{A} $$

In the formula, the friction coefficient between rock blocks is generally 0.5. R_3_ is the supporting force required to maintain the M key block.

Substituting formula (7) and formula (8) into formula (9), it can be obtained that the minimum supporting force required to maintain the stability of M key block

10 $$ R_{3} = \frac{{4i(1 - \sin \theta_{1} ) - 3\sin \theta_{1} - 2\cos \theta_{1} }}{{4i + 2i\sin \theta_{1} (\cos \theta_{1} - 2)}}P_{1} $$

In formula (10), P_1_ is the sum of the self-weight of the critical block M, R_G_, and the overburden load is subjected to R_z_^26^.

11 $$ \theta_{1} = \arcsin \frac{{L_{2} }}{{W_{1} }} $$

12 $$ P{}_{1} = R_{G} + R_{Z} $$

13 $$ R_{G} = bh_{3} L_{3} \gamma_{3} $$

14 $$ R_{Z} = K_{G} bh_{Z} L\gamma_{Z}^{{}} $$

15 $$ K_{G} = \frac{{L_{3} }}{{2h_{{_{3} }} \lambda \tan \phi }}K_{t} $$

In formulas (13) and (14), *b* is the width of the bracket; *L*_3_ is the length of the key block; γ_3_ is the bulk weight of the layered rock on the key layer; *K*_G_ is the load transfer coefficient; *h*_z_ is the thickness of the loaded layer; γ_z_ is the average capacity weight of the load layer; *K*_G_ is the load transfer coefficient when the cycle comes to pressure; K_t_ is the time factor of load transfer; the general sandy soil layer of Shendong mining area is = 27°; *K*_t_ is taken as 0.25.

In summary, the minimum supporting force to maintain the structural stability of "short masonry beams" in the sub-storey above the critical level is

16 $$ R_{3} = \left[ {\frac{{4i(1 - \sin \theta_{1} ) - 3\sin \theta_{1} - 2\cos \theta_{1} }}{{4i + 2i\sin \theta_{1} (\cos \theta_{1} - 2)}}} \right]{*}(h_{3} \gamma_{3} + K_{G} h_{z} \gamma_{Z} )bL_{3} $$

### Structural mechanical analysis of key layers at various stages

#### Structural mechanical analysis of key strata under goaf

**Figure 11:**
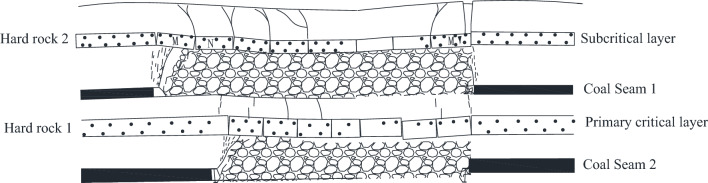
Structural model of single thick and hard key layer in upper coal seam.

Xu Jialin, Zhu Weibing, Miao Xiexing and other scholars^27–29^ classified the key layer structure of the overlying rock of the shallow buried coal seam, and put forward the concept of the thick and hard single key layer structure of the upper coal seam, the thick and hard single key layer structure of the upper coal seam has been mined refers to the existence of only one layer of hard rock in the bedrock of the two adjacent coal seams of the shallow buried deep, and the thickness and strength is larger. If the masonry beam structure formed by the roof slab is stable after the upper seam is mined back, no abnormal dynamic pressure accident generally occurs when the lower seam is mined. If the masonry beam structure formed by the overlying rock after mining of the upper coal seam is slipped and destabilized, the destabilized masonry beam can not bear the load, and all the load is applied to the rock layer between the two coal seams, which is easy to lead to the occurrence of dynamic pressure in the working face of the lower coal seam, shown in Fig. 11.

**Figure 12:**
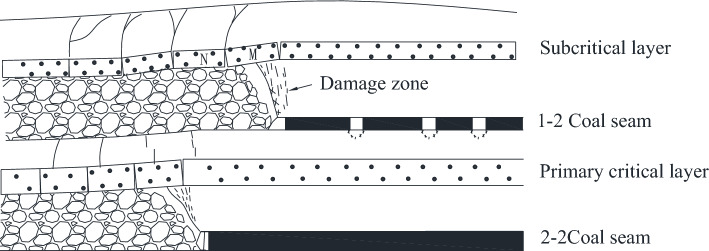
2–2 Structure diagram of overlying rock of coal seam under goaf.

**Figure 13:**
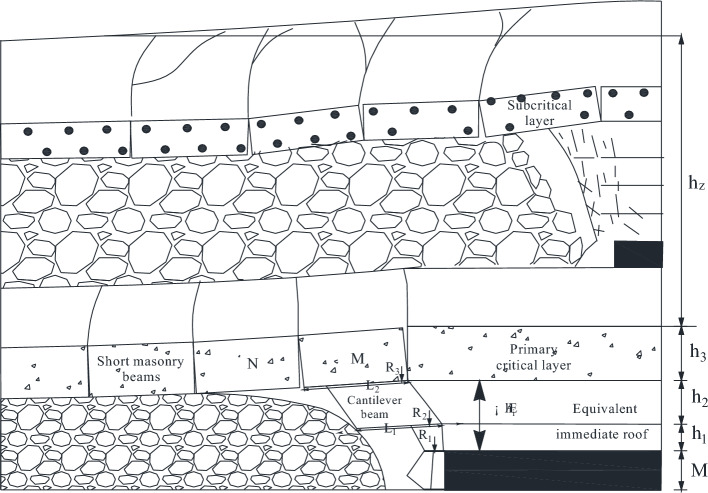
Mechanical model of roof structure of 2–2 coal seam under goaf.

When mining under the goaf of 2–2 coal seam, the sub-critical masonry beam structure in the upper part of 1–2 coal seam has slipped, and can not continue to bear the load. When 2–2 coal seam is mined, the breaking of the main key layer can not lead to the generation of strong dynamic pressure load. The structural characteristics of the stope roof before and after the mining of 2–2 coal seam are shown in Figs. 12 and 13, respectively.

**Figure 14:**
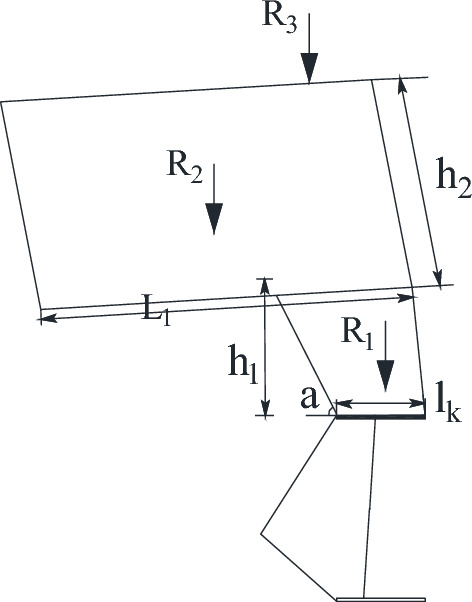
Analysis of support load.

From the above analysis, it is obvious that the sub-critical layer slips and loses stability to the goaf of 1–2 coal seam, and no longer bears the overburden load, and all the loads of overburden strata act on the rock layer between the two layers of coal. The support load is mainly composed of the equivalent direct static load and the unstable dynamic load of the "short masonry beam" structure formed by layering on the main key layer. The "short masonry beam" layered above the key layer affects the stability of the layered structure under the key layer through the instability load, and then the load is applied to the support. The mechanical model of roof structure of 2–2 coal seam under goaf shown in Fig. 13 is established. The support load is shown in Fig. 14.

The load borne by the support is as follows

17 $$ P_{m}^{{}} = R_{1} + R_{2} + R_{3} $$

In formula (17), R_1_ is the direct top dead weight; R_2_ is the force transmitted by the cantilever beam to the direct roof; the formula for calculating R_1_ and R_2_ is ^22^

18 $$ R_{1} \approx \left( {l_{k} + \frac{1}{2}h_{1} \cos \alpha } \right)bh_{1} \gamma_{1} $$

19 $$ R_{2} = bL_{1} h_{2} \gamma_{2} $$

According to formula (16), (18) and (19), the required resistance of the mining support under the goaf can be calculated as follows

20 $$ P_{m}^{{}} = \left( {l_{k} + \frac{1}{2}h_{1} \cos \alpha } \right)bh_{1} \gamma_{1} + bL_{1} h_{2} \gamma_{2} + \left[ {\frac{{4i(1 - \sin \theta_{1} ) - 3\sin \theta_{1} - 2\cos \theta_{1} }}{{4i + 2i\sin \theta_{1} (\cos \theta_{1} - 2)}}} \right]*(h_{3} \gamma_{3} + K_{G} h_{z} \gamma_{Z} )bL_{2} $$

If the support efficiency of the bracket is considered, the working resistance in the working face is

21 $$ P_{G} = \frac{{P_{m} }}{\mu } $$

According to the actual mining situation, the parameters of the formula are set to the following values: *l*_*k*_ = 6.6 m; *h*_1_ = 1.76 m; *γ*_1_ = 23.3 kN/m^3^; *α* = 60°; *L*_1_ = 8.6 m; *L*_2_ = 19.3 m; *h*_2_ = 14.93 m; *γ*_2_ = *γ*_3_ = 24 kN/m^3^; *h*_3_ = 18 m; *h*_Z_ = 48.3 m; *γ*_Z_ = 18 kN/m^3^; *K*_*G*_ = 0.48; *i* = 0.78; *θ*_1_ = 3°; *μ* = 0.9; *W*_2_ = 1 m.

By substituting the parameters into formula (20), it can be obtained that the working resistance required to control the roof in the stoping stage under the goaf is 15,023.3 kN. Considering the support efficiency of the bracket, the working resistance is 16,692.6 kN.

#### Structural mechanical analysis of inlet pillar of key layer

According to the field observation results, when the working face 22,307 advances to the lower part of the concentrated coal pillar, two cracks appear successively in the corresponding position above the main withdrawal channel, and then gradually collapse and develop into grabens. By comparing the position of the graben with the position of the working face when the coal inlet pillar is pressed, it is found that when the working face pushes to the working face 2.4 m away from the *L*_2_ fracture in the graben and strong dynamic pressure disaster occurs, the working face is 3.2 m away from the *L*_1_ fracture in the graben.

**Figure 15:**
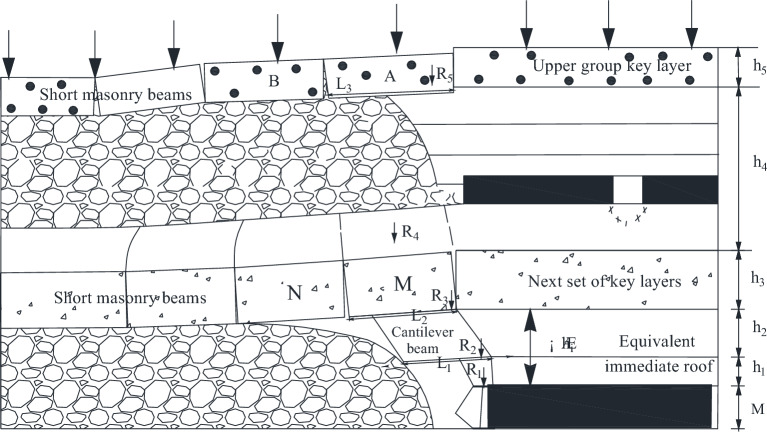
Mechanical structure diagram of "Masonry–masonry–cantilever" rock beam.

When the working face advances to the edge of the coal inlet pillar, the "masonry-masonry-cantilever" rock beam structure is established, and the mechanical model is established, shown in Fig. 15.

In Fig. 15, M is the height of coal seam 2–2; *h*_1_ is the direct top height; *h*_2_ is the layer height under the key layer; *h*_3_ is the layer height above the key layer; *L*_1_ is the fracture distance of cantilever beam; *L*_2_ is the length of the key block M of the masonry beam; *R*_1_ is the direct top weight; *R*_2_ is the force transmitted by the cantilever beam to the direct roof; *R*_3_ is the force transferred by the key block M to the equivalent direct roof; *R*_4_ is the force exerted by the inter-layer between the key layer of upper group and the key layer of lower group on the key block; *R*_5_ is the disturbed critical layer force.

**Figure 16:**
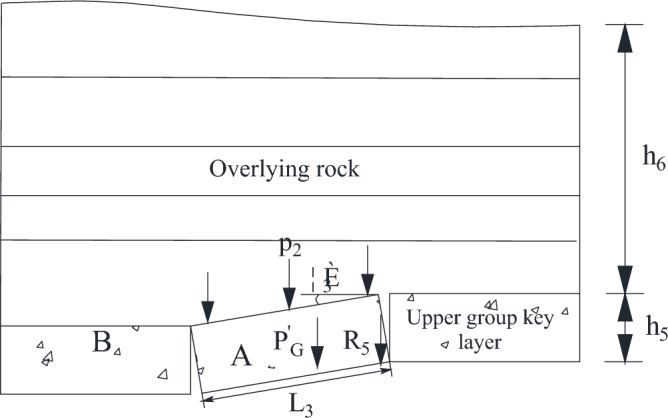
Mechanical model of disturbed key layer.

The acting force of the disturbed key layer includes the payload P_2_ transmitted by overlying strata of the disturbed key layer and the dead weight of the key block of the disturbed key layer A^30^. The mechanical model of the disturbed key layer is shown in Fig. 16.

As can be seen from the Fig. 16,

22 $$ P_{2} = K_{G}^{\prime} L_{3} \gamma_{6} h_{6}^{{}} b $$

23 $$ P^{\prime}_{G} = L_{3} \gamma_{5} h_{5} b $$

After mining the upper coal seam of shallow coal seam group, the rock block in the key layer of caving roof gradually compacts with the end of mining. In the mining process of lower coal seam, the key blocks are activated to form a "short masonry beam" structure ^31,32^. Because it is far from the working face of the lower coal seam, the rotation angle of the key block of the disturbed key layer A is small. According to the structural theory of "short masonry beam", the force of the disturbed key layer is

24 $$ R_{5} = \frac{{4i_{3} \left( {1 - \sin \theta_{3} } \right) - 3\sin \theta_{3}^{{}} - 2\cos \theta_{3} }}{{4i_{3} + 2\sin \theta_{3} \left( {\cos \theta_{3} - 2} \right)}}(P_{2} + P_{G}^{\prime } )b $$

According to Fig. 14, it can be seen that the force of the inter-layer between the upper and lower key layers is

25 $$ R_{4} = L_{2} h_{4} \gamma_{4} b $$

In summary, when 22,307 working face advances through coal pillar, the minimum support resistance required is

26 $$ P_{m} = R_{1} + R_{2} + R_{3} + R_{4} + R_{5} $$

It is obtained by substituting formulas (16), (18), (19), (24) and (25) into formula(26),

27 $$ \begin{gathered} P_{{\text{m}}} = \left( {l_{k} + \frac{1}{2}h_{1} \cos \alpha } \right)bh_{1} \gamma_{1} + bL_{1} h_{2} \gamma + \left[ {\frac{{4i(1 - \sin \theta_{1} ) - 3\sin \theta_{1} - 2\cos \theta_{1} }}{{4i + 2i\sin \theta_{1} (\cos \theta_{1} - 2)}}} \right]* \hfill \\ \left\{ {h_{3} \gamma_{3} + K_{G} \left[ {h_{4} \gamma_{4} L_{2} + \frac{{4i_{3} \left( {1 - \sin \theta_{3} } \right) - 3\sin \theta_{3}^{{}} - 2\cos \theta_{3} }}{{4i_{3} + 2\sin \theta_{3} \left( {\cos \theta_{3} - 2} \right)}}(P_{2} + P_{G}^{\prime } )} \right]} \right\}b \hfill \\ \end{gathered} $$

According to the actual mining situation on site, the parameters of the formula are set to the following values: *l*_*k*_ = 6.6 m; *h*_1_ = 1.76 m; *γ*_1_ = 23.3 kN/m^3^; *α* = 60°; *L*_1_ = 8.6 m; *L*_2_ = 19.3 m; *h*_2_ = 14.93 m; *γ*_2_ = *γ*_3_ = 24 kN/m^3^; *h*_3_ = 18 m; *h*_4_ = 14.36 m; *h*_5_ = 11.15 m; *h*_6_ = 28.33 m; *γ*_4_ = 16 kN/m^3^; *γ*_5_ = 24.6 kN/m^3^; *γ*_6_ = 20 kN/m^3^; *K*_*G*_ = 0.48; *K*_*G*_’ = 0.34; *i* = 0.78; *i*_3_ = 0.93; *θ*_3_ = 4.8°; *θ*_1_ = 3°; *μ* = 0.9.

By substituting the above parameters into formula (27), it can be obtained that the working resistance required to control the roof at the coal pillar stage is 18,021.3 kN. Considering the support efficiency of the bracket, the value is 19,692.6 kN.

#### Structural mechanical analysis of key mining layer under centralized coal pillar

**Figure 17:**
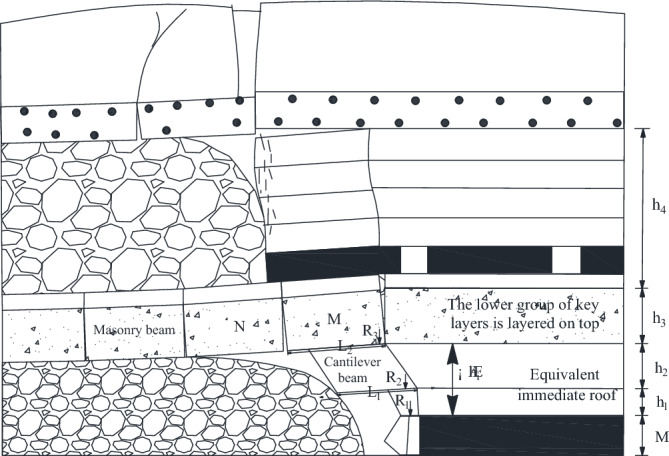
Overburden structure diagram of coal pillar stoping.

**Figure 18:**
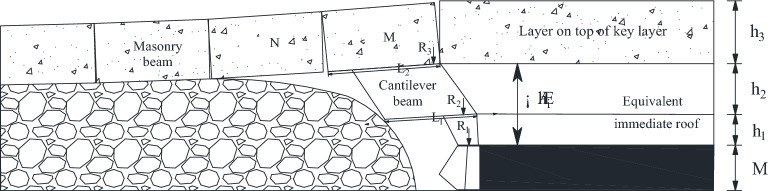
Mechanical model of mining under centralized coal pillar.

From the physical similarity simulation, it can be seen that when the 22,307 working face is mined under the concentrated coal pillar, the upper key layer forms a "cantilever beam" structure, and when the cantilever beam does not reach the breaking distance, the load cannot be transferred downward. The overlying rock structure diagram of over-concentrated coal pillar mining is shown in Fig. 17, and the mechanical model under the concentrated coal pillar is shown in Fig. 18.

The resistance of the support at this time is

28 $$ P_{m}^{{}} = \left( {l_{k} + \frac{1}{2}h_{1} \cos \alpha } \right)bh_{1} \gamma_{1} + bL_{1} h_{2} \gamma_{2} + \left[ {\frac{{4i(1 - \sin \theta_{1} ) - 3\sin \theta_{1} - 2\cos \theta_{1} }}{{4i + 2i\sin \theta_{1} (\cos \theta_{1} - 2)}}} \right]*(h_{3} \gamma_{3} + K_{G} h_{4} \gamma_{4} )bL_{3} $$

According to the actual mining situation on site, the parameters of the formula are set to the following values: *l*_*k*_ = 6.6 m; *h*_1_ = 1.76 m; *γ*_1_ = 23.3 kN/m^3^; *α* = 60°; *L*_1_ = 8.6 m; *L*_2_ = 19.3 m; *h*_2_ = 14.93 m; *γ*_2_ = *γ*_3_ = 24 kN/m^3^; *h*_3_ = 14.95 m; *h*_4_ = 14.36 m; *γ*_4_ = 16 kN/m^3^; *K*_*G*_ = 0.48; *i* = 0.78; *θ*_1_ = 3°; *μ* = 0.9.

By substituting the above parameters into formula (28), it can be obtained that the working resistance required to control the roof in the stopping stage under the centralized coal pillar is 11,835.5 kN. Considering the support efficiency of the bracket, the value is 13,150.6 kN.

#### Mechanical analysis of key layer structure during coal pillar extraction

**Figure 19:**
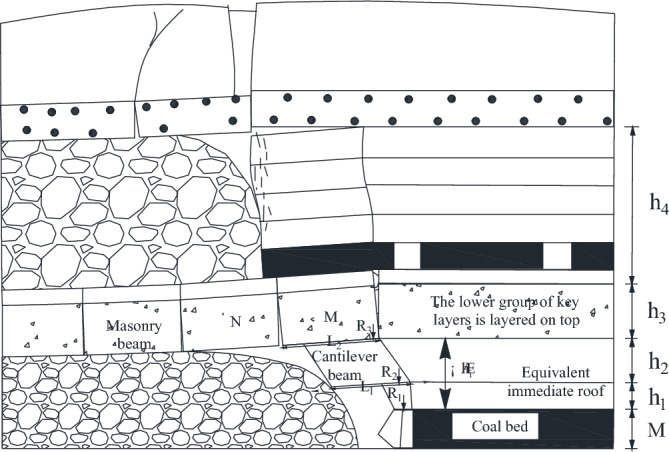
Overburden structure of coal pillar.

When the concentrated coal pillar is pushed out of the working face, the "cantilever beam" structure composed of rock strata above 1–2 coal seam reaches the breaking distance, and the overlying rock structure at this time is shown in Fig. 19.

**Figure 20:**
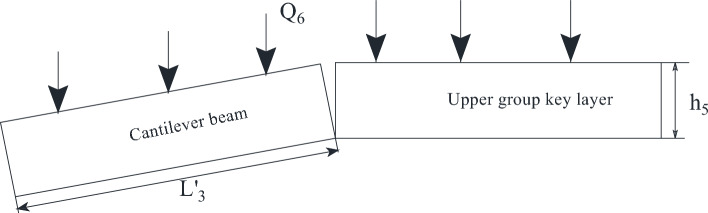
Mechanical model of cantilever beam.

In Fig. 19, Q_4_ is the load exerted on the lower key layer by the inter-layer of the upper key layer and the lower key layer; Q_5_ is the load exerted on the inter-layer by the cantilever beam fracture of the upper key layer; Q_6_ is the load imposed on the upper key layer by the rock layer between the upper key layer and the bedrock; h_4_ is the thickness of the inter-layer between the upper key layer and the lower key layer; h_5_ is the thickness of the upper key layer; h_6_ is the layer thickness between the upper key layer and the bedrock. The mechanical model diagram of the cantilever beam is established in Fig. 20.

The load imposed on the key layer in the upper group is

29 $$ Q_{6} = L^{\prime}_{3} h_{6} \gamma_{6} $$

The load exerted on the inter-layer by the cantilever beam fracture is

30 $$ Q_{5} = L^{\prime}_{3} h_{5} \gamma_{5} $$

The load imposed on the lower key layer by the inter-layer of the upper key layer and the lower key layer is

31 $$ Q_{4} = L_{{_{2} }} h_{4} \gamma_{4} $$

When the working face passes the coal pillar, the resistance of the support is

32 $$ \begin{gathered} P_{m}^{{}} = \left( {l_{k} + \frac{1}{2}h_{1} \cos \alpha } \right)bh_{1} \gamma_{1} + bL_{1} h_{2} \gamma_{2} + \left[ {\frac{{4i(1 - \sin \theta_{1} ) - 3\sin \theta_{1} - 2\cos \theta_{1} }}{{4i + 2i\sin \theta_{1} (\cos \theta_{1} - 2)}}} \right] \hfill \\ *(h_{3} \gamma_{3} + K_{G} h_{4}^{{}} \gamma_{{_{Z} }} )bL_{3} + \left( {L^{\prime}_{3} h_{6} \gamma_{6} + L^{\prime}_{3} h_{5} \gamma_{5} + L^{\prime}_{3} h_{5} \gamma_{5} } \right)b \hfill \\ \end{gathered} $$

According to the actual mining situation on site, the parameters of the formula are set to the following values: *l*_*k*_ = 6.6 m; *h*_1_ = 1.76 m; *γ*_1_ = 23.3 kN/m^3^; *α* = 60°; *L*_1_ = 8.6 m; *L*_2_ = 19.3 m; *L*_3_’ = 30 m; *h*_2_ = 14.93 m; *γ*_2_ = *γ*_3_ = 24 kN/m^3^; *h*_3_ = 18 m; *h*_4_ = 14.36 m; *h*_5_ = 11.15 m; *h*_6_ = 28.33 m; *γ*_4_ = 16 kN/m^3^; *γ*_5_ = 24.6 kN/m^3^; *γ*_6_ = 20 kN/m^3^; *K*_*G*_ = 0.48; *i* = 0.78; *θ*_1_ = 3°; *μ* = 0.9.

By substituting the above parameters into formula (32), it can be obtained that the working resistance required to control the roof at the coal pillar stage is 17,294.1 kN. Considering the support efficiency of the bracket, the value is 19,215.6 kN.

## Conclusions

This paper takes the 22,307 working face of Bulianta coal mine in Shendong as the engineering background, and adopts physical similarity simulation and theoretical analysis to study the characteristics of the overburden of shallow buried close coal seams and the mechanism of abnormal ground pressure manifestation of coal pillars at different working stages, and obtains the following conclusions.

1. According to the physical similarity simulation experiments, the 30 m thickness key layer of the roof of the 2–2 coal seam experiences layered fracture during the back-mining process of the 22,307 working face, and the lower layer of the key layer collapses into the mining area in the form of a cantilever beam structure, while the upper layer forms a masonry beam structure. When mining under the overlying centralized coal pillar, the overlying rock of the top plate of the lower group of key seams bends and sinks, causing the "masonry beam" structure formed by the top plate of the 1–2 coal seam to activate and destabilize, increasing the load of the top plate of the 2–2 coal seam, which is the main reason for the pressure frame accident and the generation of cracks in the graben.
2. When the coal pillar is discharged, the residual concentrated coal pillar and the room coal pillar are destabilized together under the action of high supporting stress, and the upper group of key layers fractures in the inner side of the coal pillar of the room pillar, and the long overhanging top and the rock layer controlled by its upper part rotates and sinks synchronously, resulting in the shear damage of the inter-layer strata in the upper part of the 2–2 coal seam. The upper and lower two groups of key layer combinations break and come to be pressurized, causing the working face to be strong and dynamic pressure to be visible, triggering the pressurized frame accident.
3. By establishing the overburden structural model and the corresponding mechanical model for each stage of back-mining (the stage of back-mining under the hollow area, the stage of entering the coal pillar, the stage of back-mining under the coal pillar, and the stage of exiting the coal pillar), the working resistance required for each stage of the support has been calculated, and the mechanism of the abnormal mine pressure in and out of the coal pillar has been reasonably explained. The final result is 16,692.6 kN of working resistance required for the bracket in the back-mining stage under the hollow area, 19,692.6 kN for the bracket in entering coal pillar stage, 13,150.6 kN for the bracket in the back-mining stage under the centralized coal pillar, and 19,215.6 kN for the bracket in exiting coal pillar stage.

## Data Availability

Some or all data, models, or codes generated or used during the study are available from the corresponding author by request.
